# Visualization of internal 3D structure of small live seed on germination by laboratory-based X-ray microscopy with phase contrast computed tomography

**DOI:** 10.1186/s13007-020-0557-y

**Published:** 2020-02-01

**Authors:** Naoki Kunishima, Yoshihiro Takeda, Raita Hirose, Dominika Kalasová, Jakub Šalplachta, Kazuhiko Omote

**Affiliations:** 1 X-Ray Research Laboratory, Rigaku Corporation, 3-9-12 Matsubara-cho, Akishima, Tokyo, 196-8666 Japan; 2 CEITEC-Central European Institute of Technology, Brno University of Technology, Purkynova 123, 612 00 Brno, Czech Republic

**Keywords:** 3D rendering, Germination, Image segmentation, Pansy seeds, Tomography, X-ray microscope

## Abstract

**Background:**

The visualization of internal 3D-structure of tissues at micron resolutions without staining by contrast reagents is desirable in plant researches, and it can be achieved by an X-ray computed tomography (CT) with a phase-retrieval technique. Recently, a laboratory-based X-ray microscope adopting the phase contrast CT was developed as a powerful tool for the observation of weakly absorbing biological samples. Here we report the observation of unstained pansy seeds using the laboratory-based X-ray phase-contrast CT.

**Results:**

A live pansy seed within 2 mm in size was simply mounted inside a plastic tube and irradiated by in-house X-rays to collect projection images using a laboratory-based X-ray microscope. The phase-retrieval technique was applied to enhance contrasts in the projection images. In addition to a dry seed, wet seeds on germination with the poorer contrasts were tried. The phase-retrieved tomograms from both the dry and the wet seeds revealed a cellular level of spatial resolutions that were enough to resolve cells in the seeds, and provided enough contrasts to delineate the boundary of embryos manually. The manual segmentation allowed a 3D rendering of embryos at three different stages in the germination, which visualized an overall morphological change of the embryo upon germination as well as a spatial arrangement of cells inside the embryo.

**Conclusions:**

Our results confirmed an availability of the laboratory-based X-ray phase-contrast CT for a 3D-structural study on the development of small seeds. The present method may provide a unique way to observe live plant tissues at micron resolutions without structural perturbations due to the sample preparation.

## Background

Finding in plant biology depends highly upon the imaging technique. Originally the plant tissues were observed using a light microscopy after a series of treatments comprising the fixing, the sectioning, and the staining. These treatments for the sample preparation often affect the native structure of organisms, thereby making the interpretation of results difficult. To achieve a live imaging of biological samples, various types of confocal microscopies were developed in these 30 years to observe a fluorescence-labeled specimen [1], including the two-photon excitation microscopy [2], the structured illumination microscopy [3], the stimulated emission depletion microscopy [4], and the photoactivated localization microscope [5]. Although these confocal microscopies allowed a time-resolved 3D-imaging of live organisms at spatial resolutions down to 30 nm and were applied to various plant tissues [6], they could visualize only labeled or autofluorescent substances within a limited thickness of samples. Recently, an optical phase-contrast tomography termed “marker-free phase nanoscopy” was developed [7], which enabled an observation of unstained specimens at 90 nm resolution but with a remaining limitation in the sample thickness.

The electron microscopy that provides a nanometer scale of spatial resolutions can be classified into the transmission electron microscope (TEM) and the scanning electron microscope (SEM) [8]. A promising application of the electron microscopy to plant specimens would be the correlative light and electron microscopy in which the light microscopy and the TEM are used in a complementary manner [9]. Another perspective is the serial block face SEM in which consecutive SEM images are integrated to reconstruct a large volume with dimensions of hundreds of microns [10, 11]. Although the electron microscopy is quite useful for the high-resolution observation, it is time consuming and requires a laborious preparation of ultrathin specimens with thicknesses of around 100 nm, due to a low penetration power of electron beams.

The simplicity in the sample preparation is important not only for the accessibility but also for keeping the native structure of specimens. For that reason, the X-ray microscopy with the computed tomography (CT) technique emerged as another methodology for a non-destructive 3D-imaging from much thicker plant tissues without staining by contrast reagents. However, because the unstained biological samples absorb hard X-rays only weakly, available contrasts from an absorption-contrast CT image are poor in general. Therefore, to enhance the limited contrast in the X-ray microscopy, a phase-retrieval technique was developed utilizing the much higher contribution of the phase to the contrast when compared to that of the absorption in the energy region of hard X-rays [12–16]. This technique retrieves the phase information based on a propagation-based X-ray imaging that measures fringes appeared at the boundaries of weakly absorbing materials in original projection images depending upon the sample-to-detector distance [17]; the phase-retrieved projection images are used to reconstruct a phase-contrast CT image with the better contrast. For instance, the phase-contrast CT using synchrotron X-rays has been applied to visualize a live maize seed [18]. Recently, a laboratory-based X-ray microscope adopting the phase-retrieval technique has been developed using in-house X-ray sources. This laboratory-based X-ray phase-contrast CT allows the visualization of internal 3D-structure of certain animal specimens at micron resolutions without any staining by contrast reagents [19–22], although its applications to plant specimens are limited.

Here we introduce the observation of an unstained small seed using the laboratory-based X-ray phase-contrast CT. The X-ray microscope apparatus used in this work is commercially available as nano3DX (Rigaku, Tokyo, Japan) that implements a high-brilliance X-ray generator and a high-resolution detector, thereby enabling the propagation-based phase retrieval. To date, a few observations in X-ray radiographies have been reported on dry seeds from certain plants including *Arabidopsis thaliana* [23] and sugar beet [24]. The X-ray phase-contrast imaging has been applied to visualize overall root-soil interactions [25]. Recently, certain plant specimens have been observed using the laboratory-based X-ray phase-contrast CT [26, 27]. However, wet seeds during the germination have never been observed due to their low absorption contrast. In this work, the phase-retrieval technique was successfully applied to enhance the weak contrast of unstained wet seeds. To this end, a pansy (*Viola* × *wittrockiana*) seed was selected as a model because of its suitable size with dimensions of about 2 × 1 × 1 mm^3^ and its hard/smooth texture suppressing local motions. To our knowledge, this is the first report on the observation of germinating seeds using the laboratory-based X-ray microscope.

## Results

### Observation of pansy seed

**Fig. 1:**
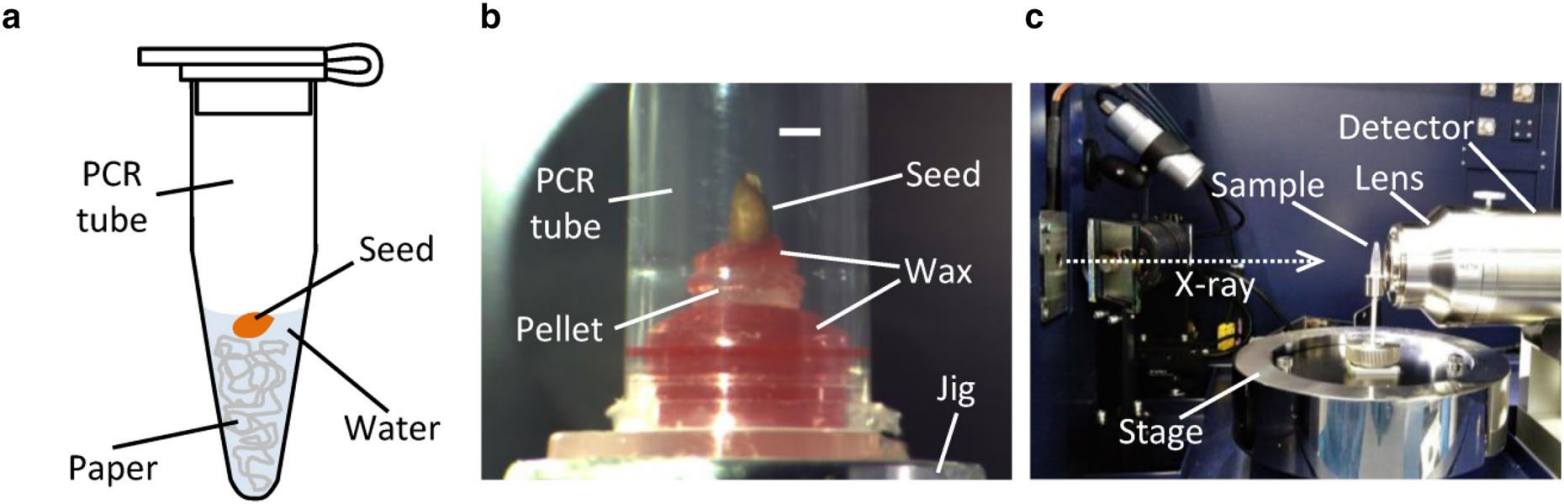
Observation of pansy seed. **a** Watering of a seed. A pansy seed incubated with water in a 0.2 ml PCR tube is schematically represented. **b** Photograph of a dry seed mounted for X-ray scanning. Scale bar: 1 mm. **c** Configuration of X-ray scanning. This photograph represents a setting for the high-resolution imaging as described in “Methods” section

We observed live pansy seeds using a laboratory-based X-ray microscope. For the observation of a pansy seed during the germination, the seed was incubated in advance with water for various duration of soaking in a PCR tube (Fig. 1a). Then a dry seed or the wet seed taken out from the tube was mounted for the X-ray scanning (Fig. 1b). The seed was fixed carefully on the sample stage of the microscope, because the CT reconstruction was susceptible to a sample drift during the data collection. Fixing the specimen with a wax in a capped PCR tube was found as a successful method. In the case of a wet seed, a small amount of water was placed beside the specimen to avoid an evaporation-induced deformation. The seed was irradiated by in-house X-rays from a Cu-target to collect projection images for the CT reconstruction (Fig. 1c).

### Contrast enhancement

**Fig. 2:**
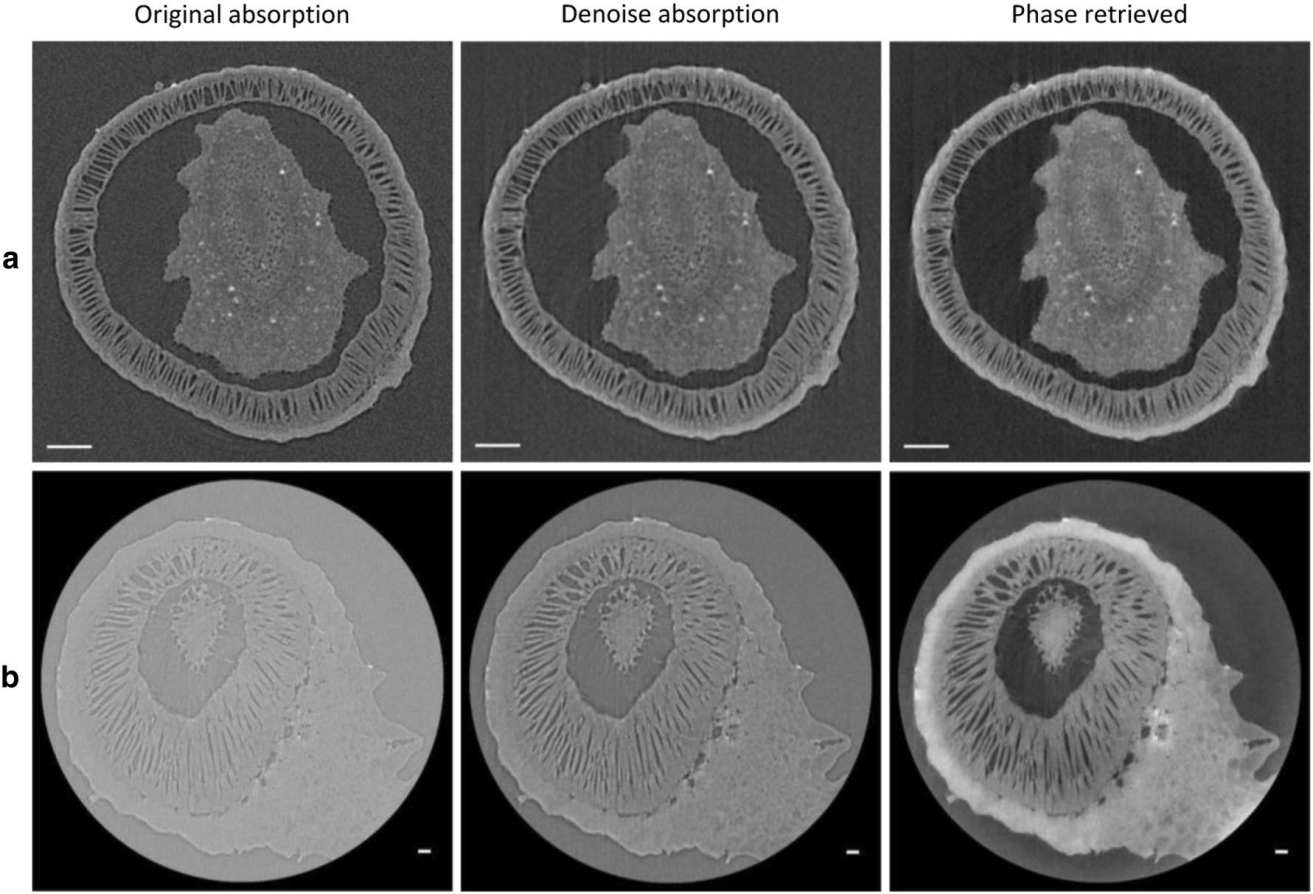
Contrast enhancement. Corresponding CT slices from three different treatments are displayed. A dry seed was directly mounted on the tip of a metal rod (φ 3 mm × 55 mm) with a double-sided tape and the rod was set on the sample stage of the nano3DX-sCMOS with the Cu-target. **a** Low resolution observation. For the data collection, 1000 frames with a 1.44 s exposure per frame were taken by the continuous-scan mode with a voxel size of (1.27 μm)^3^ (L1080 lens, bin 1, sample-to-detector distance of 4 mm). **b** High resolution observation. For the data collection, 300 frames with a 2.40 s exposure per frame were taken by the continuous-scan mode with a voxel size of (0.63 μm)^3^ (L0270 lens, bin 2, sample-to-detector distance of 4 mm). Scale bars: 100 μm in **a** and 20 μm in **b**

To enhance the weak contrast in the projection images from unstained seeds, a phase-retrieval technique based on the Paganin’s method [13] has been employed with a δ/β coefficient of 600 assuming that the sample was made of organic compounds (Table 1 and Fig. 2). The phase retrieval successfully provided a remarkable enhancement in the signal-to-noise ratio (SNR) of output CT slices. For a comparison, the projection images were treated alternatively by a conventional median/Gaussian-based noise reduction (denoise) before the CT reconstruction. Notably, the fringes seen in the absorption-contrast images are disappeared in the phase-retrieved images with improved SNRs. In the present work, the boundary fringes from which phases were retrieved were about several microns in size, since the sample-to-detector distance was set at 4 or 7 mm. Pairwise *t*-tests confirmed a significant difference between any combination of average SNR values in the three CT slices from the same projection data set (*p* < 0.001). Then we analyzed the spatial resolution of the images. Pairwise *t*-tests confirmed a significant difference between any combination of average resolution values in the three CT slices from the same projection data set (*p* < 0.001), except for *p* = 0.019 between the original absorption and the denoise absorption of the low-resolution observation and *p* = 0.43 between the original absorption and the denoise absorption of the high-resolution observation. Thus, the phase retrieval apparently deteriorates the resolution of images, indicating a trade-off relationship between the SNR and the resolution, although this deterioration in resolution is thought to be canceled by considering the size of boundary fringes. Importantly, the higher resolution provided the better SNR. Therefore, a submicron size of voxel may be required to obtain the best result in the laboratory-based X-ray phase-contrast CT. This tendency may be relevant to a limited size of the fringes, and therefore may be specific to the laboratory-based X-ray microscopy adopting a quasi-parallel beam with short sample-to-detector distances. In case of the synchrotron radiation, much longer distances by the meter are used [18], indicating the lower dependency upon resolution.

**Table 1:** Statistics of contrast enhancement

	SNR	Resolution (μm)
Low resolution (n = 15)		
Original absorption	3.44 ± 0.24	1.63 ± 0.14
Denoise absorption	8.78 ± 1.34	1.86 ± 0.11
Phase retrieved	13.15 ± 1.52	2.25 ± 0.15
High resolution (n = 5)		
Original absorption	1.39 ± 0.10	0.93 ± 0.17
Denoise absorption	4.27 ± 0.58	1.03 ± 0.18
Phase retrieved	18.04 ± 1.39	1.62 ± 0.12

A projection data set collected at low or high resolution was treated by three different methods before the CT reconstruction, and the three corresponding CT slices were analyzed. The SNR value was calculated as an average ± 95% CI from indicated numbers of comparisons between background and compound areas of 10 pixels × 10 pixels each for the low-resolution images and of 16 pixels × 16 pixels each for the high-resolution images, according to the definition described in “Methods” section. The resolution value was calculated as an average ± 95% CI from indicated numbers of line profiles of 25 μm for the low-resolution images and of 10–15 μm for the high-resolution images, according to the procedure described in “Methods” section

### Segmentation of embryo

**Fig. 3:**
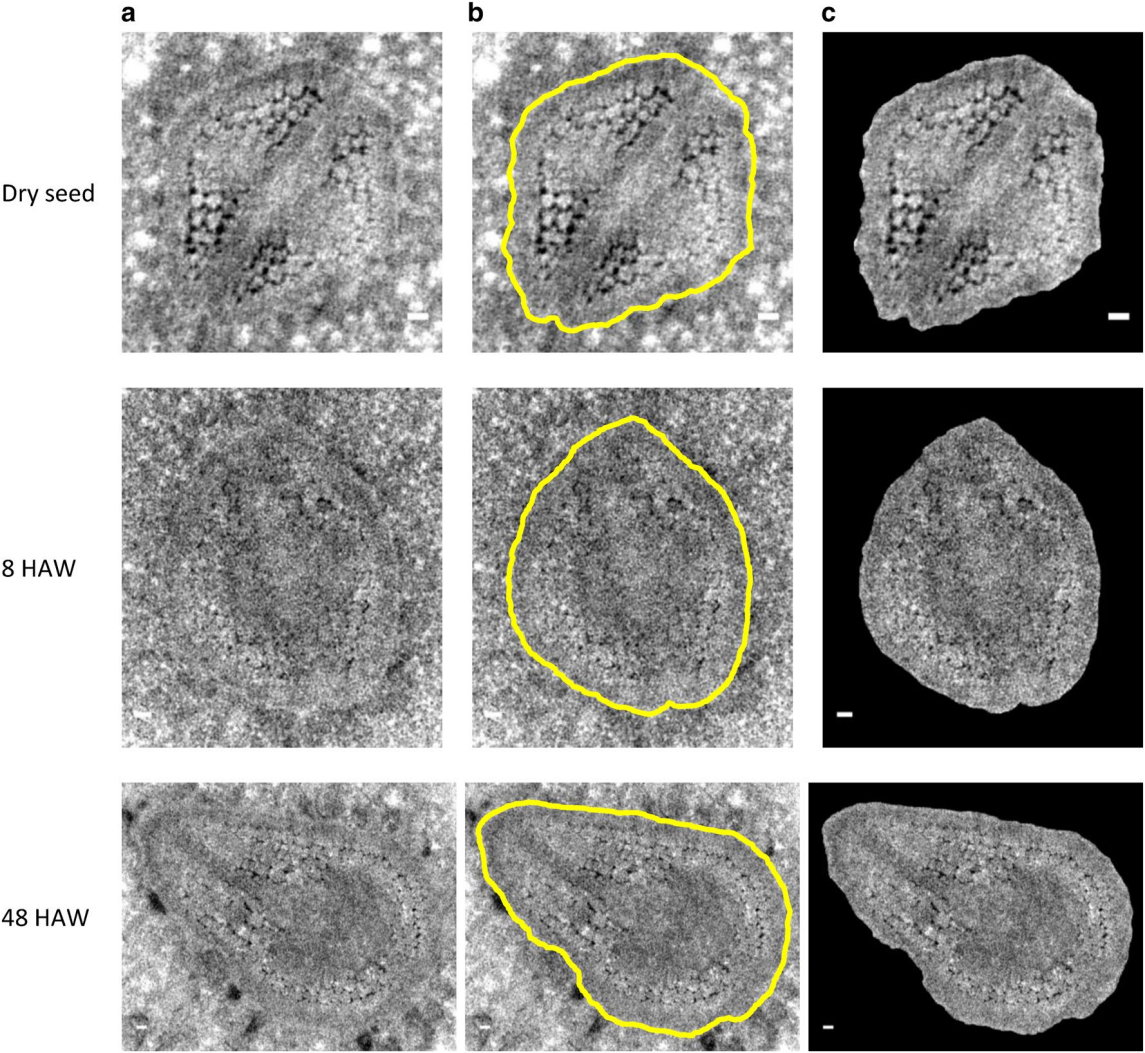
Segmentation of embryo. The manual segmentation procedure is explained using high-resolution CT slices of pansy seeds at three stages of germination; the data used are the same as those used in Fig. 4. **a** Original CT slices magnified around embryos. **b** Corresponding CT slices with lines that delineate embryo boundaries. **c** Corresponding embryos segmented. Scale bars: 20 μm

By a combination with the phase-retrieval technique, the laboratory-based X-ray microscopy enabled a manual segmentation of pansy embryos from high-resolution CT slices (Fig. 3a). In the manual segmentation, an embryo was delineated utilizing relatively high densities in the boundary area (Fig. 3b). The SNR values between the boundary and the neighboring areas were in the range of 0.30–0.55. This is comparable to the reported SNR of 0.45 for the manual segmentation of a maize embryo [18]. In the segmented embryos of both the dry and the wet seeds, comprising cells were clearly resolved (Fig. 3c), suggesting the availability of the laboratory-based X-ray phase-contrast CT for the cellular level observation of unstained live plant tissues.

### Structure of pansy seed at three stages of germination

**Fig. 4:**
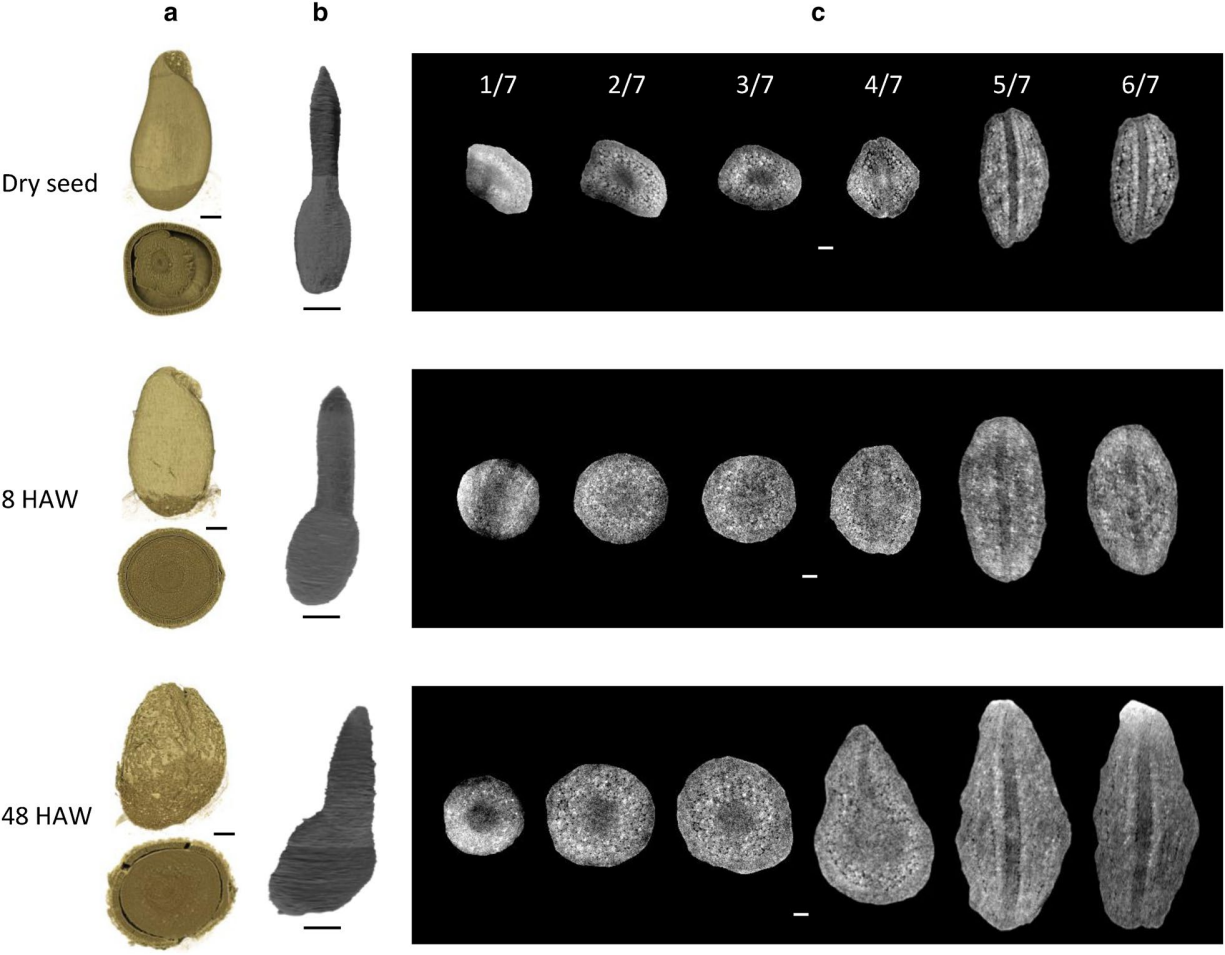
Structure of pansy seed at three stages of germination. The experiment procedure is described in “Methods” section. Seeds at three different duration of watering are shown as 3D rendering models produced using the program *Drishti* [36]. **a** Outlines (top) and cross-sections (bottom) of whole seeds from low-resolution observations. **b** Whole embryos segmented from high-resolution observations. Lateral streaks on the embryo surface are artifacts due to a technical limitation of the manual segmentation. **c** Six transverse slices that equally divide an embryo in **b** from the top to the bottom. Relative positions of the slices are indicated in the dry-seed panel. The perspective is from the right side in **b**. Scale bars: 300 μm in **a**, **b** and 50 μm in **c**

As an application of the present method, we tried to observe the development of pansy embryos during the germination. Unfortunately, a time-course observation from a single seed was unsuccessful because the X-ray dose in one time of the data collection killed the seed irradiated. Furthermore, the variation of seeds hampered a detailed analysis of morphological changes. Then, in this work, we observed three representative stages of germination from different seeds with typical morphologies. For the dry seed, three seeds were observed and the most typical one was represented. We tried various duration of watering from 30 min to 24 h and selected a typical one at 8 h after the start of watering (8 HAW). A germinated seed was selected from three seeds at 48 h after the start of watering (48 HAW). From a low-resolution observation, an overall morphological development of seeds during the germination process was visualized (Fig. 4a). The low water content of the dry seed makes internal matters shrunk to form a large void space; the dryness provides the best contrast in the CT image, as expected. At 8 HAW, the internal matters swell to occupy the void space. The germinated seed exhibit clacks on the seed coat probably due to the enlargement of the embryo at 48 HAW. On the other hand, from a high-resolution observation, a morphological development of embryos during the germination process was clarified by the manual segmentation (Fig. 4b). The cotyledons and the base of the primordial root grow rapidly during the germination, which produces a curvature on the overall shape of the embryo. This distortion of the embryo may induce the clacking of the seed coat when the germination occurs. Corresponding to the overall morphological development, the cotyledons and the base of the primordial root grow rapidly during the germination (Fig. 4c). Notably, the growth between the dry seed and 8 HAW seems to be a simple swelling by water, whereas that between 8 and 48 HAW seems to be induced by substantial enlargement in the cortex of the root base as well as in the whole of the cotyledon.

### Internal 3D-structure of pansy seed

**Fig. 5:**
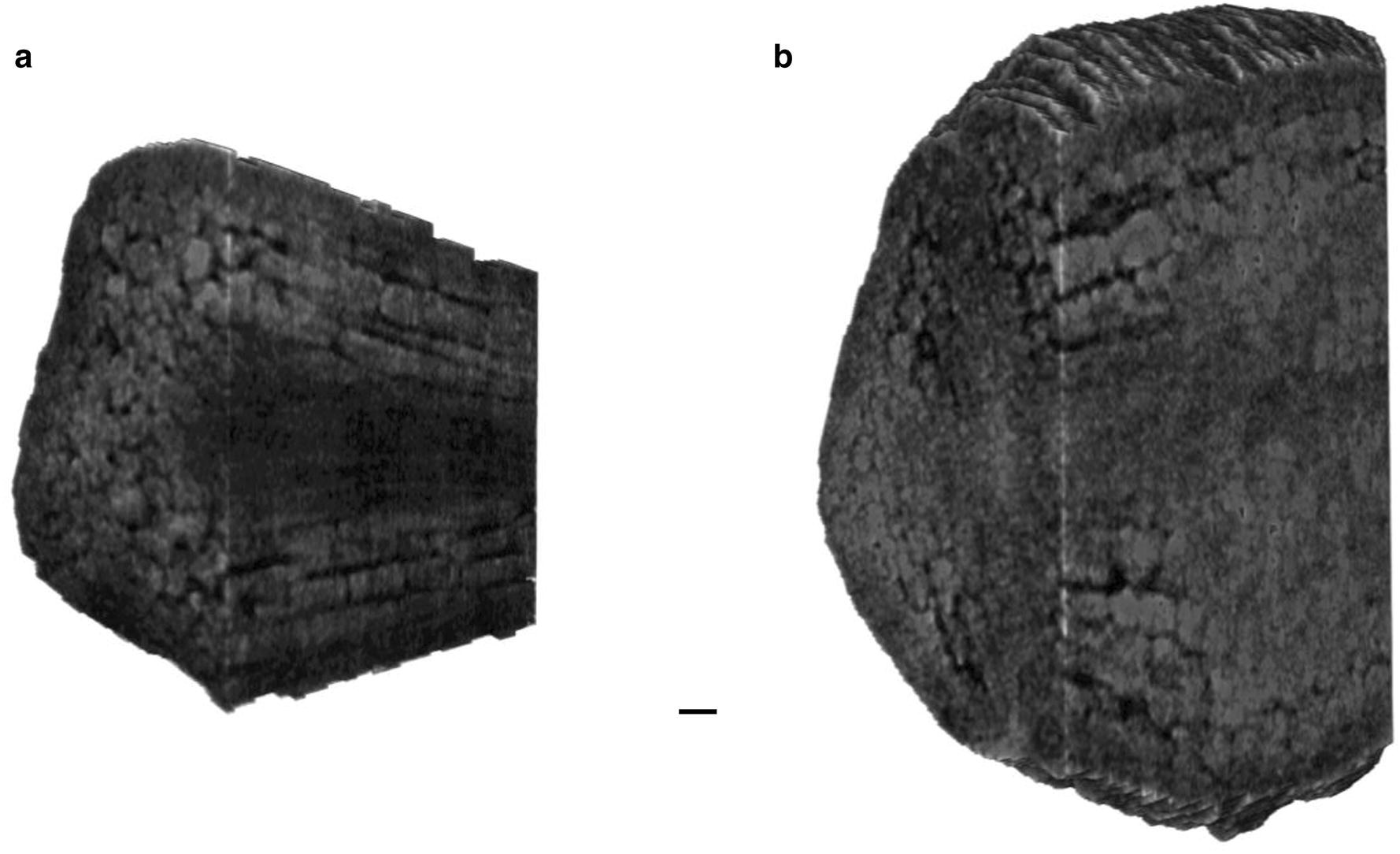
Internal 3D-structure of pansy seed. Two 3D-rendering models produced by *Drishti* are represented for the intervening CT slices **a** 2/7–3/7 and **b** 4/7–5/7 of a dry seed as shown in Fig. 4c. The models are tilted to the left by 30° to show longitudinal sections. Lateral grooves on the embryo surface are artifacts due to a technical limitation of the manual segmentation. Scale bar: 20 μm

The internal 3D-structure of a dry seed was further analyzed using a 3D-rendering technique. In the primordial root of the dry seed embryo, cells are longitudinally arranged to make a cortex that will be developed to the vascular bundle in a mature plant (Fig. 5a). The other parts including the medulla and the periphery show the lower density probably reflecting a difference of chemical contents. In the cotyledon, cells are less aligned when compared to those in the root, and they make another cortex that will be developed to the parenchyma in the mature plant (Fig. 5b). The primordial leaf vein can be seen as a low-density extension from the root medulla. The base region of the cotyledon shows the higher density probably indicating the meristem of the plant. This putative meristem looks enlarged in the later stages of the germination (Fig. 4c). Wet seeds also revealed similar internal 3D-structures in their 3D renderings, although their contrasts are lower when compared to those in the dry seed (Additional file 1: Fig. S1). Because of the limited contrast in the hydrated embryos, we could not perform further analyses on the cell development, unfortunately.

## Discussion

In this study, we report the observation of unstained pansy seeds using a laboratory-based X-ray microscope. The phase-retrieval technique was adopted to enhance the contrast of the projection images. From a dry seed, 300 phase-retrieved projection images from a fifteen minutes exposure in total were enough to reconstruct a tomogram revealing complex internal 3D-structures at a cellular level; the embryo could be extracted manually from the tomogram. Wet seeds also provided tomograms with enough contrasts for the segmentation of embryos from 600–700 phase-retrieved projection images, suggesting an applicability of present method to analyze the embryo development during the germination of small seeds. However, the X-ray CT method used in this work was destructive to the live seeds, unfortunately. Thus, another method enabling a time-course observation of a single germinating seed would be desirable. If a limited number of projection images can sketch out the seed structure at any germination stage, a time-resolved X-ray projection method may be worth trying in future.

In this work, a manual segmentation was used to extract embryo from the CT images. As a result, the minimum SNR required for the manual segmentation was found to be 0.30 when the embryo boundary and the neighbor regions were compared; the phase retrieval was essential to achieve the requirement. An automatic segmentation may be desirable to facilitate the segmentation in future. To date, a typical SNR required for the automatic segmentation using the active contour method [28] is around 1.5 in the case of a maize embryo [18]. Although the pansy seed used in this work had relatively high germination rate of 60%, variation of seeds made the morphological analysis difficult. Searching for seeds from the other organisms with the better homogeneity may help the analysis.

The laboratory-based X-ray microscopy with the phase-retrieval technique may be useful to observe unstained biological specimens at micron resolutions. Currently, this method is applicable to the tissue/cellular level observation of plant specimens. The phase retrieval showed a trade-off relationship between the SNR and the resolution. This relationship may be investigated further in future using certain statistical analyses such as the response surface method [29] and the kernel density estimation [30, 31]. Further improvement of the spatial resolution is required to expand the applicability to the subcellular level. This subcellular level observation at submicron resolutions may require the more advanced technology to fix the cellular motions during the data collection, like the cryo-protection in the soft X-ray tomography [32, 33].

## Conclusions

In light of the results, the laboratory-based X-ray microscopy with the phase-retrieval technique may be useful to observe unstained biological specimens at micron resolutions, which may contribute to solve the major problem in plant researches.

## Methods

### Plant material

In this study, we used commercially available seeds of pansy (*Viola* × *wittrockiana*) that were purchased from a manufacturer (Atariya Nouen, Chiba, Japan). The watering to a dry seed was performed at room temperatures around 24 °C by adding a 45 μl aliquot of regular water on a piece of paper (KimWipe S-200; Nippon Paper Crecia, Tokyo, Japan) with dimensions of about 7 mm × 35 mm that was stuffed in a 0.2 ml polypropylene PCR tube with a flat cap (Fig. 1a). The light was not controlled. Under this condition in the capped PCR tube, the germination occurred on about 60% of seeds at 48 h after the start of watering (48 HAW).

### Data collection

A pansy seed (about 2 × 1 × 1 mm^3^ in size) was fixed with a wax (Utility Wax; GC Corporation, Tokyo, Japan) on a cylindrical plastic pellet (2.8 × 2.8 × 2.0 mm^3^ in size), and the pellet was fixed in the PCR tube at the inner side of the cap; the hinge of the cap was cut off to avoid its clash with the detector (Fig. 1b). The outer side of the flat cap of the PCR tube was fixed on a cylindrical metal jig (12 × 12 × 12 mm^3^ in size) with a double-sided tape, and the jig was set on the sample stage of an X-ray microscope apparatus (Fig. 1c): nano3DX with a scintillator-based lens and with a 16 bit 2048 × 2048 sCMOS detector (Rigaku, Tokyo, Japan). To achieve the propagation-based imaging as well as to reduce the influence from a drift of the light source, the sample-to-detector distance was set much shorter than the source-to-sample distance (260 mm) so as to produce a quasi-parallel X-ray beam. In the case of wet seeds, about 10 μl aliquot of regular water was placed beside the plastic pellet in the capped PCR tube to prevent the evaporation. The seed mounted was irradiated by in-house X-rays from a Cu-target (8.0 keV of energy; 1.54 Å of wavelength) to collect projection images with a continuous-scan mode. For an overall imaging of a seed without the phase retrieval, 800 projection images with a 1.50 s exposure per frame were collected with a voxel size of (1.25 μm)^3^ (L1080 lens, bin 1, sample-to-detector distance of 7 mm); the absorption contrast was enhanced by a conventional median/Gaussian-based noise reduction (denoise). For a high-resolution imaging with the phase retrieval to extract the embryo from a seed, two datasets from the top and the bottom side of the seed were merged using the software *ImageJ* [34] so as to cover the entire of the embryo; for a dataset from a dry seed or from a wet seed of 8 HAW, 600 projection images with a 1.60 s exposure per frame were collected with a voxel size of (0.63 μm)^3^ (L0540 lens, bin 1, sample-to-detector distance of 7 mm); for a dataset from a wet seed of 48 HAW, 700 projection images with a 1.58 s exposure per frame were collected with the same camera setting.

### Phase retrieval and reconstruction

To enhance the contrast of the high-resolution projection images, the phase retrieval was performed based on the Paganin’s method [13] with the δ/β coefficient of 600. The CT reconstruction at 16 bit was performed based on a conventional filtered back projection method implemented in ASTRA Toolbox [35]. The 3D rendering was performed using the program *Drishti* [36]. The segmentation of the embryo was performed manually using the free-hand selection tool in *ImageJ*, as follows. In each CT slice, the boundary of the embryo was delineated, and the selected area of embryo was isolated from the other area by the “Clear Outside” tool. Consecutive slices with the isolated area of embryo were combined to reconstruct a whole embryo. The selection of the previous slice was copied to the current slice when the positional difference between the two embryo boundaries was small; the boundary was redrawn when the positional difference exceeded by about 10 μm.

### Analysis of images

The images were analyzed from two aspects. For each analysis, a 95% confidence interval (CI) of the average value was calculated to clarify its accuracy. The statistical significance on the difference between a pair of average values was evaluated by the Student’s *t*-test under a null hypothesis of no difference; the equality of variances was confirmed in advance by the *F*-test; the Welch’s *t*-test was applied in the case of unequal variances. A signal-to-noise ratio (SNR) between two regions of an image was calculated as follows:

1 $$SNR = \frac{{\left| {\mu _{1} - \mu _{2} } \right|}}{{\sqrt {\sigma _{1}^{2} + \sigma _{2}^{2} } }} ,$$

where *μ*_1_ and *μ*_2_ represent the average density values of the two regions and *σ*_1_ and *σ*_2_ represent their corresponding standard deviations; only homogeneous regions were used for the calculation. The spatial resolution of an image was estimated from a curve fitting with a four-parameter logistic function against a line density profile across a well-defined edge in the image:

2 $$y = A - \frac{{A - B}}{{1 + \left( {{\raise0.7ex\hbox{$x$} \!\mathord{\left/ {\vphantom {x C}}\right.\kern-\nulldelimiterspace} \!\lower0.7ex\hbox{$C$}}} \right)^{D} }},$$

where variables *x* and *y* represent the position and the value of a pixel, respectively, and the parameters *A* to *D* represent the maximum asymptote value, the minimum asymptote value, the inflection position, and the Hill’s slope, respectively. A distance between two positions giving values *A* − 0.25(*A* − *B*) and *A* − 0.75(*A* − *B*) was defined as the spatial resolution; only the distance greater than the pixel size was accepted.

## Supplementary information

**Additional file 1: Figure S1.** Internal 3D-structure of pansy seed at three stages of germination.

## Data Availability

Data and materials used in the present study are available from the corresponding author on reasonable request.

## Abbreviations

CI: Confidence interval
CT: Computed tomography
SNR: Signal-to-noise ratio
