# Supplementary material for: Visualization of internal 3D structure of small live seed on germination by laboratory-based X-ray microscopy with phase contrast computed tomography
Source: Plant Methods. 2020 Feb 1;16:7. doi: 10.1186/s13007-020-0557-y (PMC6995115; doi:10.1186/s13007-020-0557-y)
Supplement: Supplementary file 1 — Additional file 1: Figure S1. Internal 3D-structure of pansy seed at three stages of germination. [file 13007_2020_557_MOESM1_ESM.pdf]

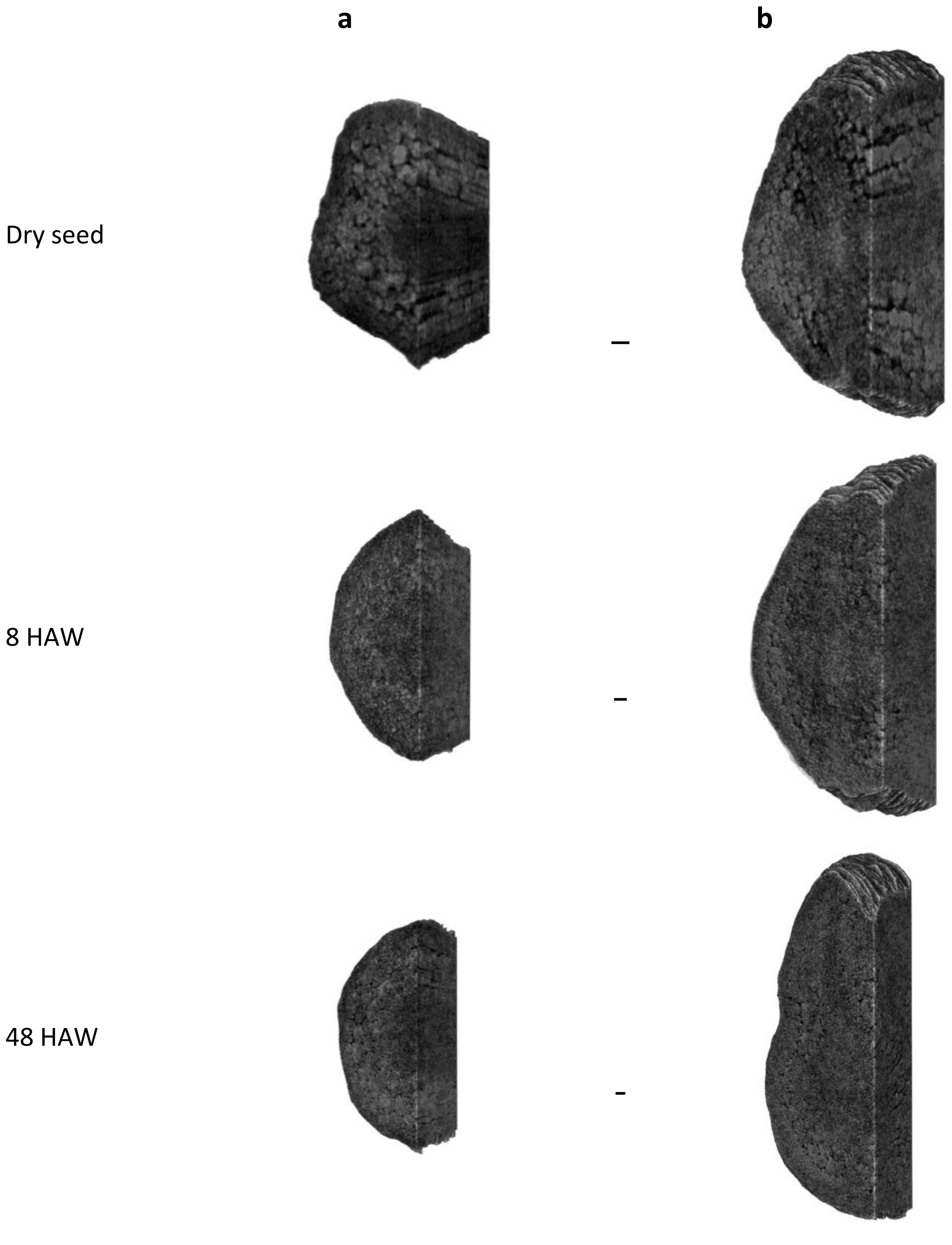

Fig. S1 Internal 3D-structure of pansy seed at three stages of germination.
Two 3D-rendering models produced by *Drishti* are represented for 220 CT slices **a** from 2/7 toward 3/7 and **b** from 4/7 toward 5/7 of seeds at three different stages of the germination as shown in Fig. 4c. The models are tilted to the left by 30° to show longitudinal sections. Lateral grooves on the embryo surface are artifacts due to a technical limitation of the manual segmentation. Scale bars: 20 μm
